# One Health surveillance integrating human, mosquito, and climate data for early warning of chikungunya outbreaks in East Africa

**DOI:** 10.1016/j.nmni.2026.101868

**Published:** 2026-10-09

**Authors:** Mohamed Abdulkadir Hussein, Ilyas Abdullahi Khalif, Saida Abdirzak Mohamed, Salma Zubair Mohamud, Hibo Abdullahi Mohamed

**Affiliations:** a Faculty of Health Sciences and Tropical Medicine, Somali National University, Mogadishu, Somalia; b Center for Health Research and Innovation, Somali National University, Mogadishu, Somalia; c Banadir Research, Consultancy & Evaluation Center, Mogadishu, Somalia

**Keywords:** Chikungunya, One health, Surveillance, *Aedes*, Climate change

Dear Editor.

Chikungunya surveillance in East Africa remains too reactive because it is still anchored largely to recognition and reporting of human illness, even though chikungunya was first identified in East Africa, continues to recur across Africa, and increasingly spreads through urban and peri-urban human–mosquito transmission rather than only sylvatic spillover [1]. This dependence on clinical case detection is intrinsically delayed by inapparent infections, overlap with other febrile illnesses, and limited standardization of surveillance, all of which leave outbreaks sporadic, unpredictable, and often recognized only after amplification is underway (see Table 1).

**Table 1:** Selected documented chikungunya case numbers and surveillance findings across East Africa.

Country	Year	Location	Suspected Cases	Confirmed Cases	Total Reported Cases	Deaths	Vector	Source
Kenya	2004	Lamu Island, coastal Kenya	not reported	not reported	not reported	not reported	not reported	[2]
Kenya	2016	Mandera City	not reported	not reported	not reported	not reported	*Aedes aegypti/Aedes albopictus* reported as primary East African vectors	[1], [2]
Tanzania	2007–2008	not reported	not reported	not reported	not reported	not reported	*Aedes aegypti/Aedes albopictus*	[1], [2]
Uganda	2000–2026	data not reported in supplied corpus	not reported	not reported	not reported	not reported	*Aedes aegypti/Aedes albopictus*	[1]
Ethiopia	2019	Dire Dawa	41,162	16	41,162 suspected + 16 laboratory-confirmed	not reported	*Aedes aegypti*	[3]
Somalia	2016	first cases, location not reported	not reported	not reported	not reported	not reported	*Aedes aegypti/Aedes albopictus*	[1]
Burundi	2000–2026	data not reported in supplied corpus	not reported	not reported	not reported	not reported	*Aedes aegypti/Aedes albopictus*	[1]
South Sudan	2000–2026	data not reported in supplied corpus	not reported	not reported	not reported	not reported	*Aedes aegypti/Aedes albopictus*	[1]
Djibouti	2019–2020	Djibouti City	not reported	58 in French Defense Community outbreak surveillance	58 in FDC surveillance cohort	not reported	*Aedes aegypti*	[3]
Djibouti	2019–2020	Djibouti City, national urban outbreak	not reported	not reported	not reported	not reported	*Aedes aegypti*	[3]

The case for reform is stronger under climate change. Climatic suitability for mosquitoes that transmit chikungunya is increasing, with warmer conditions expanding geographic range and lengthening transmission seasons [1], [4]. Experimental and field evidence indicates that temperature, rainfall, and humidity shape *Aedes* abundance and transmission potential: higher temperatures generally increase mosquito infection and transmission efficiency, rainfall increases *Aedes* abundance, and precipitation and temperature extremes are strong predictors of habitat suitability and hotspot formation. Recent outbreak analyses also show that heavy rainfall and high temperatures can rapidly generate high vector densities and accelerate local spread, while broader climate-event cascades interact with urbanization, land use change, and mobility to intensify outbreak risk [4].

These dynamics expose the weakness of passive human surveillance alone. Entomological studies show that vector infection prevalence can be low and focal even when arboviruses are circulating, meaning that the absence of detected virus in sampled mosquitoes does not imply the absence of local risk [5]. At the same time, vector surveillance can still provide actionable early signals through adult female density, egg counts, larval habitat mapping, vector distribution, and risk stratification, which help identify areas with active transmission potential before clinical notifications peak. Recent work further shows that combining entomological, epidemiological, and environmental data supports targeted vector control and resource prioritization.

A One Health early warning architecture is therefore more appropriate for East Africa than siloed surveillance. One Health surveillance explicitly integrates human, animal, and environmental intelligence and, crucially, includes surveillance of drivers of emergence rather than pathogens alone [6]. Operational examples from climate-sensitive vector-borne disease programs show that integrating clinical and laboratory diagnosis with vector, environmental, epidemiological, and genomic surveillance can reveal overlooked transmission clusters, track viral diversity, and guide preventive action. Regional networks have also shown that long-term multisectoral collaboration strengthens diagnostic capacity, vector monitoring, risk assessment, and coordinated response across borders [7].

## 1 East African chikungunya case evidence

Available peer-reviewed evidence provides exact outbreak counts for Kenya, Ethiopia, and Djibouti, but for Tanzania, Uganda, Somalia, Rwanda, Burundi, and South Sudan [3].

The largest documented outbreaks in the supplied East Africa corpus are in Ethiopia, especially Dire Dawa in 2019, reported as 41,162 suspected cases with 16 laboratory-confirmed cases in one outbreak report and as over 50,000 suspected cases in a regional review using WHO-AFRO-linked surveillance synthesis [2]. Kenya clearly had major epidemics in 2004 and again in 2016–2018, but the supplied papers mostly describe timing, locations, and attack rate rather than exact case totals; Lamu Island reached a 75% attack rate in 2004 [2]. Djibouti has a confirmed 2019–2020 urban outbreak, but the exact citywide extent was not available in the supplied peer-reviewed paper, which explicitly notes missing dedicated reporting; only the French Defense Community surveillance subset reported 58 confirmed cases [3].

The main periods of increased transmission were the 2004–2005 East African resurgence centered on Kenya, the 2007–2008 Tanzania activity, and the 2016–2020 wave affecting Mandera, Mombasa, Somalia, Ethiopia, and Djibouti [1], [2]. Outbreaks cluster in coastal, urban, and transport-linked settings such as Lamu, Mombasa, Kilifi, Dire Dawa, and Djibouti City, consistent with transmission by *Aedes aegypti* and, in some settings, *Aedes albopictus* or other locally relevant *Aedes* species [1]. Mombasa entomology also found CHIKV in *Aedes* vittatus, while *Aedes aegypti* remained the dominant collected vector.

The major surveillance gaps are that several countries in the requested list have serologic evidence or mention of circulation without extractable outbreak counts, and reviews explicitly note disparities between reported cases, genomic data, and dedicated reporting systems [2]. Tanzania and Ethiopia studies also emphasize inadequate laboratory capacity, under-recognition, and geographic heterogeneity in detection. These patterns support integrated early warning that combines human case surveillance, mosquito surveillance focused on *Aedes aegypti* and *Aedes albopictus*, and climate-sensitive monitoring because temperature and rainfall shape vector abundance and transmission risk.

Across the requested East African countries, the strongest case-number evidence in the supplied literature is for Ethiopia, Kenya, and Djibouti, while substantial numeric surveillance gaps remain for Uganda, Somalia, Rwanda, Burundi, and South Sudan.

East Africa faces exactly the constraints that integrated surveillance is designed to overcome: fragmented sectoral systems, weak data sharing, uneven laboratory capacity, and transboundary arboviral risk in a region where mobility, urban growth, and climate variability are reshaping exposure [6]. The policy implication is clear. Ministries of health should move from passive case reporting toward regionally coordinated One Health surveillance platforms that link routine human case detection with mosquito monitoring and climate intelligence, supported by interoperable data systems, shared laboratory protocols, and cross-border alert thresholds. In East Africa, earlier chikungunya detection will depend less on counting cases after the spread begins and more on integrating the signals that precede them.

## CRediT authorship contribution statement

**Mohamed Abdulkadir Hussein:** Conceptualization. **Ilyas Abdullahi Khalif:** Methodology. **Saida Abdirzak Mohamed:** Writing – original draft. **Salma Zubair Mohamud:** Writing – review & editing. **Hibo Abdullahi Mohamed:** Writing – review & editing.

## Ethics approval and consent to participate

Not applicable. This is based exclusively on previously published literature and does not involve human participants or primary data collection.

## Consent for publication

Not applicable.

## Availability of data and materials

Not applicable.

## Clinical trial number

Not applicable.

All authors have reviewed and approved this declaration.

## Funding

This research received no external funding.

## Declaration of competing interest

The authors declare that they have no known competing financial interests or personal relationships that could have appeared to influence the work reported in this paper.
